# Unlocking hope: domino liver transplantation for maple syrup syndrome, a single center experience work carried out at the King Fahad Specialist Hospital

**DOI:** 10.3389/fimmu.2025.1579945

**Published:** 2025-05-01

**Authors:** Mohammed Al Qahtani, Rawan Aloufi, Ethar Gaw, Hammam Momani, Bodhisatwa Sengupta, Iftikhar Khan, Ibrahim Hassan, Razan Bader, Rehab Abdullah, Mansour Tawfeeq, Ahmed Zidan

**Affiliations:** ^1^ Organ Transplant Center of Excellence, King Fahad Specialist Hospital, Dammam, Saudi Arabia; ^2^ Surgery Department, Assiut University, Assiut, Egypt

**Keywords:** domino liver transplant, pediatric liver transplant, liver transplant, domino living donor liver transplant, living donor liver transplant (LDLT)

## Abstract

**Background:**

Maple Syrup Urine Disease (MSUD) is an autosomal recessive disorder caused by mutations in the BCKDH complex, leading to the accumulation of branched-chain amino acids. Severe cases of MSUD often require liver transplantation (LT) to restore metabolic stability and prevent neurological complications. Domino liver transplantation (DLT) using MSUD livers has emerged as an innovative approach to expand the donor pool, leveraging the fact that MSUD-affected livers can function normally in recipients without developing MSUD due to extrahepatic BCKDH activity.

**Methods &Results:**

This study retrospectively reviews the experience at King Fahad Specialist Hospital in Dammam, where seven patients with MSUD underwent LT, with their explanted livers subsequently transplanted into seven other recipients. The results demonstrate the feasibility and safety of this approach, with a 100% survival rate for MSUD patients at a median follow-up of 2.9 years. For the domino recipients, the 3-year graft and patient survival rate was 71.4%, with two graft-related fatalities.

**Conclusion:**

The study highlights the importance of careful recipient selection, optimal graft-to-recipient weight ratio, and the potential for hybrid dual graft transplantation in cases where graft volume is insufficient. The findings suggest that DLT using MSUD livers is a viable option, particularly in regions with limited deceased donor activity, and should be considered in mature liver transplant programs to address organ shortages.

## Introduction

Maple Syrup Urine Disease (MSUD) is recognized as an autosomal recessive monogenic inherited disorder characterized by mutations in the branched-chain ketoacid dehydrogenase (BCKDH) complex. These mutations result in the loss of function of this enzyme complex, leading to the accumulation of branched-chain amino acids (BCAAs) such as leucine, isoleucine, and valine (1, 2).

The consequences of MSUD can be severe, encompassing neurological disabilities, coma, brain edema, and even mortality. While dietary management can mitigate symptoms, severe cases often necessitate liver transplantation to provide a permanent solution and prevent neurological complications (3, 4).

Liver transplantation (LT) emerges as a paramount therapeutic strategy for MSUD due to the liver’s pivotal role in accounting for 9% to 13% of the body’s total BCKDH production. Through LT, restoration of BCAA homeostasis becomes feasible, thereby mitigating the risk of enduring neurological sequelae (1, 5, 6).

Notably, the liver procured from a donor with MSUD maintains both structural and functional integrity, rendering it suitable for use as a graft in domino liver transplantation (DLT). DLT represents a logical approach aimed at expanding the donor pool, with recipients of an MSUD domino graft being spared from developing the disease, as BCAA metabolism occurs predominantly in extrahepatic tissues, notably the kidneys and muscles (7, 8).

By amalgamating existing literature with our institutional experience at King Fahad Specialist Hospital (KFSH) in Dammam, encompassing seven patients who underwent domino liver transplantation from MSUD liver grafts, this report endeavors to furnish invaluable perspectives on the role of MSUD liver grafts as a source of, underscoring its potential to confer enduring metabolic correction, elucidating its feasibility outcome.

## Method

Between November 2009 and December 2023, our center performed 471 liver transplant, of which 276 (58.5%) were living donor liver transplants, including 106 (22.5%) pediatric liver transplants for recipients under 18 years old. Notably, 10 of these pediatric cases were diagnosed with maple syrup urine disease (MSUD), with 7 patients subsequently underwent domino liver transplants utilizing livers from individuals with MSUD at King Fahad Specialist Hospital in Dammam.

Data pertaining to both donors and recipients were meticulously gathered through a combination of retrospective examination of medical records and the maintenance of a prospectively collected database, all conducted with prior approval from the hospital’s ethics committee.

In evaluating potential recipients with MSUD, our standard protocol involved a thorough assessment, including computed tomography with volumetry to measure the liver volume and assess the vascular anatomy. Notably, three MSUD livers were deemed unsuitable for domino liver transplantation due to either insufficient graft volume or complex vascular anatomy.

Of the seven MSUD donors, six received left lateral segment (LLS) liver grafts from living donors, while one received a full liver graft from a deceased donor. Once suitability of the MSUD liver was confirmed in operation room, simultaneous exploration of the second recipient were initiated, with the liver being perfused with University of Wisconsin (UW) solution and maintained on ice until the hepatectomy of the second recipient was completed. Subsequently, the liver was flushed with albumin before implantation, and venoplasty was performed on the back table, involving the approximation of the right hepatic vein stump with the stump of the middle and left hepatic veins confluence to create a single orifice for anastomosis.

In the second recipient, the right hepatic vein and the confluence of the middle and left hepatic veins were connected together with a downward extension into the inferior vena cava, forming a generous orifice that was subsequently joined to the hepatic veins of the graft as a single anastomosis. Standard procedures were followed for portal vein and hepatic artery anastomosis, with duct-to-duct anastomosis or hepaticojejunostomy being performed based on the size of the duct on the donor side.

## Results

Between November 2009 and December 2023, seven MSUD patients underwent liver transplantation, six of which were living donor liver transplants. The explanted MSUD livers were subsequently transplanted into seven recipients as domino allografts.

The median age of the MSUD recipients was 8.4 years, with a range from 2 to 13 years. All MSUD patients, except one, received living donor liver transplantation. The median age of the domino liver transplantation (DLT) recipients was 50.8 years, ranging from 40 to 61 years. The mean MELD score at transplantation for the MSUD liver (domino) recipients was 20.4, with a median of 17 and a range of 15 to 34. The diverse diagnoses among the recipients, which included hepatocellular carcinoma (HCC), non-alcoholic steatohepatitis (NASH), hepatitis B virus (HBV), and alcoholic liver disease (ALD), highlight the broad applicability of domino liver transplantation.

All the MSUD patients received the left lateral segment as a living donor liver transplant, except for one who received a full liver graft as a deceased donor liver transplant. The median graft weight was 189 grams, ranging from 195 to 380 grams (**Table 1**). The table shows our standard surgical technique for patients who received a living donor graft.

**Table 1:** Demographic data for MSUD patients and MSUD graft recipient.

	MSUD Patient			MSUD graft recipient			
	Age (y)	Weight (Kg)	Liver weight (gm)	Age (y)	Weight (Kg)	Diagnosis	MELD
1	12	28.45	554	55	56.6	HCC	17
2	5	18	499	54	61.3	NASH	13
3	12	39.4	531	48	48.8	HBV	24
4	13	37.7	693	41	86	NASH+HCC	34
5	7	23	607	57	69.5	NASH	23
6	8	21.9	542	61	72.25	NASH	17
7	2	13.6	468	40	74.3	ALD	15

For venous anastomosis, the left hepatic vein (LHV) of the left lateral segment (LLS) was connected to the LHV of the recipient, and the left portal vein of the graft was anastomosed to the main portal vein of the recipient. For arterial reconstruction, the left hepatic artery of the graft was anastomosed to the common hepatic artery of the recipient. Finally, biliary reconstruction was achieved with a hepaticojejunostomy (**Table 2**).

**Table 2:** Intra operative data of MSUD patients.

	Type of the graft	Weight of the graft	Hepatic venous anastomosis	Portal Vein anastomosis	Hepatic artery anastomosis	Biliary anastomosis
1	Full Liver	380	IVC to IVC	MPV to MPV	CHA to CHA	D-D
2	LLS	195	LHV to LHV	LPV to MPV	LHA to CHA	H-J
3	LLS	245	LHV to LHV	LPV to MPV	LHA to CHA	H-J
4	LLS	230	LHV to LHV	LPV to MPV	LHA to CHA	H-J
5	LLS	220	LHV to LHV	LPV to MPV	LHA to CHA	H-J
6	LLS	315	LHV to LHV	LPV to MPV	LHA to CHA	H-J
7	LLS	312	LHV to LHV	LPV to MPV	LHA to CHA	H-J

In our series, we strictly adhered to the principle that the safety and optimal outcome of the primary (MSUD) recipient take precedence over considerations for the secondary (domino) recipient. The technical aspects of the donor operation were not altered to accommodate the domino liver transplantation. This practice aligns with the widely accepted standard that prioritizes the primary recipient’s safety, even if it results in a domino graft that cannot be utilized. This approach is consistent with the principle outlined by Soltys et al., emphasizing that placing additional risk on the primary recipient to optimize the domino graft is not justified (9).

In this study, the graft-to-recipient weight ratio (GRWR) ranged from 0.79 to 1.08, with cold ischemia time (CIT) varying between 94 and 283 minutes, and warm ischemia time (WIT) ranging from 22 to 45 minutes. Hepatic venous anastomosis was achieved using same technique, the right hepatic vein (RHV) and the confluence of the middle hepatic vein (MHV) and left hepatic vein (LHV) were approximated with venoplasty and anastomosed to the confluence of the three hepatic veins in the recipient. In patient 7, who has a unique situation will be discussed later the hepatic veins of the graft were anastomosed to the MHV/LHV confluence in the recipient.

Portal vein anastomosis was consistently performed from the main portal vein (MPV) of the graft to the MPV of the recipient, except in patient 7, where the MPV was anastomosed to the right portal vein (RPV) of the recipient. Arterial anastomosis varied, with most patients receiving anastomosis from the common hepatic artery (CHA) to the right hepatic artery (RHA), while in patient 2, the CHA was connected to the right gastroepiploic artery, and in patient 4, a jump graft was used to connect the CHA to the infrarenal aorta. Biliary reconstruction was performed with duct-to-duct (D-D) anastomosis in all cases (**Table 3**).

**Table 3:** Intraoperative data of MSUD graft recipient.

	GRWR	CIT (min.)	WIT (min.)	Hepatic venous anastomosis	Portal Vein anastomosis	Hepatic artery anastomosis	Biliary anastomosis
1	0.97	94	45	RHV and confluence of MHV/LHV are approximated with venoplasty and anastomosed to the confluence of the 3 HVs in the recipient	MPV to MPV	2 separate anastomosis to RHA&LHA	D-D
2	0.81	195			MPV to MPV	CHA with right gastroepiploic artery	D-D
3	1.08	240	22		MPV to MPV	CHA to RHA	D-D
4	0.8	240	30		MPV to MPV	CHA to infrarenal aorta with jump graft	D-D
5	0.87	269	36		MPV to MPV	CHA to RHA	D-D
6	0.79	283	29		MPV to MPV	CHA to CHA	D-D
7	0.91*	236	32	The HVs of the graft anastomosed to the MHV/LHV confluence in the recipient	MPV to RPV	CHA to RHA	D-D

In the patient number 7, we have innovated scenario, we encountered a situation where the domino graft volume for the recipient was insufficient, prompting the utilization of a domino liver graft as part of a dual graft liver transplant by using LLS from living donor to augment the liver volume to meet the metabolic demands of the recipient, innovating what is called Hybrid dual graft liver transplant using domino and Living donors.

For MSUD patients the patient and graft survival are 100% at current median follow up 2.9 years ranging from 5–96 months. Two patients had developed Clavien-Dindo grade III complications; one had hepatic artery thrombosis in post operative day 5 which required re exploration and revision of the hepatic artery, the other one developed bile leak which required ultrasound guided drainage and the leak resolved conservatively.

For MSUD graft recipient, 3-year patient and graft survival are 71.4%.Patient number 4, the graft reperfusion after portal vein anastomosis was very slow and mosaic, next day after transplant, his lactate continue to rise up, His transaminases were >2000 and the INR was more than 9 in a picture suggesting primary nonfunctioning graft and he passed away in the same day. Patient number 5 developed a severe chest infection on post operative day 12 which required re intubation and then had developed clostridial difficile infection in the colon and he eventually deteriorated and developed septic shock and died on day 42 post-transplant. The remaining 5 survivors are doing well with follow up, only one has developed biliary stricture which required ERCP and stenting, all of them on regular follow up assessment of Valine, leucin and isoleucine levels on annual base and none of them required any diet modifications.

## Discussion

Domino liver transplant for MSUD patients represents an innovation in the field of liver transplantation, offering a unique dual benefit. Firstly, it serves as a classical management strategy tailored to address the complex needs of MSUD patients. Moreover, domino liver transplantation also emerges as an essential tool in mitigating the persistent challenge of graft shortage within the transplantation community specially in countries with low deceased donor activity (6, 8, 10, 11).

In essence, domino liver transplant for MSUD patients not only represents a significant advancement in the personalized treatment of metabolic disorders but also serves as a strategic solution to enhance the efficiency and accessibility of liver transplantation services on a broader scale (12).

In our series, All except one of the donors for MSUD patients have been living donors who donated left lateral segments (LLS). This reflects the prevailing situation not only in our hospital but also throughout the entire region, where LDLT serves as the primary source of liver transplants (13, 14). However, this scenario presents a significant challenge.

Specifically, in LDLT, the vessels of the liver grafts from living donors tend to be shorter compared to those from deceased donor grafts. This discrepancy in vessel length necessitates careful consideration and balancing between the length of the vessels in MSUD patients and those that will be retained in the MSUD liver graft for domino transplant in another recipient. Navigating this delicate balance requires meticulous planning and surgical expertise to optimize outcomes for both the MSUD patients and MSUD liver recipients (15, 16).

Another pivotal factor in the success of domino liver transplantation for MSUD patients is the careful selection of domino liver graft recipients. Given that the domino liver is deficient in BCKDH enzyme activity, it is imperative to choose recipients who are free from any kidney or muscle diseases, as they will serve as the main source of BCKDH enzyme production. However, there have been no reports of *de novo* disease appearance in DLT patients with MSUD livers, since the liver accounts for only approximately 9%-13% of BCKDH activity in the entire body (7, 8, 11, 17). Our findings indicate that MSUD patients can be safe and effective liver donors for candidates with long-term life expectancy. Instead of being viewed as bridge grafts for a future normal allograft, MSUD patients can provide viable and durable donor livers.

Pediatric candidates are well-described for this option since DLT serves as an excellent alternative to deceased donor allografts, given the difficulty of finding a size-matched organ in this patient population. However, none of our recipients were pediatric (18). This could be because we are adopting the concept of reserving the domino grafts for patients who have no available living donor and those who have been on the waiting list for a long time. Since our primary liver transplant activity involves living donors, most of our potential pediatric recipients are presented with potential living donors.

Several studies have highlighted the advantages of selecting low MELD recipients for domino liver grafts to optimize outcomes (8, 17, 19, 20). In our study, the average MELD score of the recipients was 20, and the average age was 50 years old. This approach is rooted in theoretical hypotheses suggesting that despite the normal function and structure of the domino liver grafts except for the deficiency in BCKDH enzyme activity, the liver could potentially be affected by the metabolic crises experienced by the MSUD donor. In the recipient number 4, his MELD was relatively high 34 with significant amount of signs of portal hypertension which could be a reason that he could not survive such a marginal domino graft.

In our series of DLT in MSUD patients, we have underscored two critical considerations that have been pivotal in guiding our approach. Firstly, we prioritize ensuring that the graft volume of the donor liver destined for MSUD patients is sufficient, ideally around a GRWR of 1. This emphasis is crucial as the transplanted liver becomes the sole source of the BCKDH enzyme in the recipient’s body. Maintaining a robust BCAA hemostasis without the need for a restricted diet hinges upon the adequacy of the graft volume. Therefore, achieving an optimal GRWR ratio plays a pivotal role in securing the metabolic stability and long-term health of MSUD patients post-transplantation.

Secondly, paramount importance is placed on prioritizing the safety and well-being of both MSUD patients and their potential donors. This principle takes precedence over the desire to obtain a favorable domino liver graft for subsequent transplantation in another recipient. While the concept of utilizing the liver from an MSUD patient as a domino graft holds promise in expanding the donor pool, ensuring the safety of all individuals involved remains paramount. Thus, careful consideration and rigorous evaluation are undertaken to mitigate any potential risks to both the MSUD patient and the prospective donor.

Despite the technical and logistical challenges in DLT, in patient 7, we have expanded the use of domino livers by adding a left lateral segment (LLS) from a living donor as a dual graft liver transplant. This approach helps meet the metabolic demands of the recipient and avoids small-for-size syndrome. Hybrid dual graft liver transplantation using domino and living donors should be incorporated into the armamentarium of a mature live donor liver transplant and domino liver transplant center. It has been shown to facilitate the completion of the procedure while prioritizing safety for both the liver donor and the domino graft recipient, resulting in acceptable outcomes (21).

Many studies have reported 1-year patient survival rates in MSUD graft recipients ranging from 66.7% to 100% (7, 12, 17, 20). Our study demonstrated a 3-year patient and graft survival rate of 71.6%, with only one death attributed to graft-related reasons. The variance in the outcome in the studies may be related to many factors either related to graft itself or the factors related to the recipient, but some studies have reported the effect of cold ischemia time (CIT) on the graft survival which is logistically challenges as it required working of multiple surgical teams simultaneously which could lower the CIT, this logistic challenges is better managed in centers doing living donor liver transplant as they have the ability to overcome such a challenge (11, 15, 19).

Despite the relatively low number of DLT cases in our series, which may have limited impact on reducing the overall waiting list, this option can be crucial in countries with low deceased donor activity. For patients without available living donors, DLT offers a viable alternative that can potentially save lives. In regions where deceased donor organs are scarce, DLT can serve as a critical lifeline, providing an essential solution for those in need of transplantation. Therefore, the adoption of DLT using MSUD livers should be considered an important component of liver transplant programs, especially in areas with low deceased donor rates.

## Conclusion

DLT using MSUD livers is a safe and viable option for liver transplantation with acceptable outcomes. However, it should be performed in centers with mature liver transplant programs. Although the number of DLT procedures is generally low, it can be the only solution for patients without available living donors, especially in regions with low deceased donor activity.

## Data Availability

The original contributions presented in the study are included in the article/supplementary material. Further inquiries can be directed to the corresponding author/s.
